# Novel isolates of hydrogen-oxidizing chemolithoautotrophic *Sulfurospirillum* provide insight to the functions and adaptation mechanisms of Campylobacteria in shallow-water hydrothermal vents

**DOI:** 10.1128/msystems.00148-24

**Published:** 2024-08-21

**Authors:** Li Wang, Xinyi Cheng, Yizhe Guo, Junwei Cao, Mingye Sun, Jiang-Shiou Hwang, Rulong Liu, Jiasong Fang

**Affiliations:** 1 College of Oceanography and Ecological Science, Shanghai Ocean University, Shanghai, China; 2 Institute of Marine Biology, National Taiwan Ocean University, Keelung, Taiwan; 3 Laboratory for Marine Biology and Biotechnology, Qingdao National Laboratory for Marine Science and Technology, Qingdao, China; University of East Anglia, Norwich, United Kingdom

**Keywords:** *Sulfurospirillum*, hydrogen-oxidizing, Campylobacteria, shallow-water hydrothermal vents, Chemoautolithotrophic

## Abstract

Enhancing the availability of representative isolates from hydrothermal vents (HTVs) is imperative for comprehending the microbial processes that propel the vent ecosystem. In recent years, Campylobacteria have emerged as the predominant and ubiquitous taxon across both shallow and deep-sea vent systems. Nevertheless, only a few isolates have been cultured, primarily originating from deep-sea HTVs. Presently, no cultivable isolates of Campylobacteria are accessible in shallow water vent systems (<200 m), which exhibit markedly distinct environmental conditions from their deep-sea counterparts. In this study, we enriched a novel isolate (genus *Sulfurospirillum*, Campylobacteria) from shallow-water HTVs of Kueishan Island. Genomic and physiological analysis revealed that this novel Campylobacteria species grows on a variety of substrate and carbon/energy sources. The pan-genome and phenotypic comparisons with 12 previously isolated *Sulfurospirillum* species from different environments supported the identification of functional features in *Sulfurospirillum* genomes crucial for adaptation to vent environments, such as sulfur oxidation, carbon fixation, biofilm formation, and benzoate/toluene degradation, as well as diverse genes related with signal transportation. To conclude, the metabolic characteristics of this novel Campylobacteria augment our understanding of Campylobacteria spanning from deep-sea to shallow-water vent systems.

**IMPORTANCE:**

Campylobacteria emerge as the dominant and ubiquitous taxa within vent systems, playing important roles in the vent ecosystems. However, isolated representatives of Campylobacteria have been mainly from the deep-sea hydrothermal fields, leaving a significant knowledge gap regarding the functions, activities, and adaptation strategies of the vent microorganisms in shallow-water hydrothermal vents (HTVs). This study bridges this gap by providing insights into the phenomics and genomic diversity of genus *Sulfurospirillum* (order Campylobacterales, class Campylobacteria) based on data derived from a novel isolate obtained from shallow-water HTVs. Our mesophilic isolate of *Sulfurospirillum* not only augments the genus diversity of Campylobacteria pure cultures derived from vent systems but also serves as the inaugural reference isolate for Campylobacteria in shallow-water environments.

## INTRODUCTION

Hydrothermal vents (HTVs), where geothermally heated water is expelled through fissures in Earth’s crust, constitute one of the most productive marine ecosystems. As hydrothermal fluid rises from the deep crust, a variety of chemicals and volatile gases enriched in the fluid escape to the surrounding seawater. This mixing of the expelled fluid and surrounding seawater occurs both below and above the seafloor, spanning vast distances. Chemolithoautotrophs within the vent system, including animal symbionts, microbial mats members, and free-living cells, utilize various reductive substrates (such as H_2_, H_2_S, CH_4_, and Fe^2+^) as energy sources to fix carbon (1, 2). Subsequently, this organic carbon sustains dense animal communities through symbiotic relationships with bacteria, as well as through grazing, suspension feeding, and subsequent trophic transfer. While vent ecosystems in the deep-sea (>200 m) primarily rely on microbial chemosynthesis (3), shallow-water hydrothermal vents (SW-HTVs) at depths <200 m exhibit distinct environmental conditions characterized by the presence of light, terrestrial inputs, tidal cycles, and meteoric water inputs. These factors contribute to the formation of highly diverse and complex microbial communities (4, 5).

Campylobacteria (formerly known as Epsilonproteobacteria) (6) are commonly detected chemoautotrophic taxa in vent systems. Despite the discovery of deep-sea HTVs in 1977, Campylobacteria have been identified as the dominant bacteria through molecular studies since the late 20th century (7–9). They have been found in various biomes of vent systems, including chimneys, subsurface, diffuse flow, fluids, or even as symbiont of metazoans (1, 2). Campylobacteria can comprise over 80% of microbial communities in the chimney structures (10, 11), diffuse flow vent emissions (12), and over 90% in subsurface sediment (13) and plume (14). Cultivation breakthrough have revealed their metabolic versatility, with sulfide and hydrogen gas as electron donors, and elemental sulfur and nitrate as electron acceptors for lithotrophic growth (15, 16). Phylogenetically diverse Campylobacteria representatives have been isolated from deep-sea HTVs, including genera *Sulfurovum* (17–21), *Sulfurimonas* (22–25), *Caminibacter* (26), *Cetia* (27), *Lebetimonas* (28), *Nautilia* (29–31), *Nitratiruptor* (32), *Thioreductor* (33), and *Hydrogenimonas* (34, 35). While *Sulfurovum* and *Sulfurimonas* belong to the order Campylobacterales, others are from the order Nautiliales, comprising moderately thermophilic bacteria. Unlike Nautiliales, most Campylobacterales representatives thrive at lower temperatures and tolerate a wider range of oxygen concentrations (15). In SW-HTVs, Campylobacteria have been identified as dominant bacteria in fluids and sediments with moderate to high temperatures (35–55°C) and high concentrations of hydrogen sulfide and other reduced sulfur compounds, but no Campylobacteria have been isolated from SW-HTVs (5). Considering the significant environmental differences between SW-HTVs and deep-sea HTVs (4, 5), bacteria in SW-HTVs may exhibit unique functions and adaptations compared with their deep-sea counterparts. Successful cultivation of Campylobacteria isolates from SW-HTVs may shed light on their ecological roles and adaptation mechanisms to shallow water vent systems.

Kueishan Island is a young volcanic island situated at the southernmost part of the Okinawa Trough of the western Pacific Ocean, housing a series of SW-HTVs off its coast. The hydrothermal fluid and volcanic gases emanating from the Kueishan Island SW-HTVs contain high concentrations of CO_2_ and H_2_S (36). Various sulfur formations, such as sulfur chimneys, sulfur sands, and sulfur balls, are widely distributed on the seabed (37). Previous studies have consistently identified Campylobacteria as the dominant taxa in the total bacterial community based on DNA or RNA analyses (38–43). In our study, we successfully isolated multiple strains of Campylobacteria from the Kueishan Island SW-HTVs using optimized isolation medium. These isolates were classified as novel species within the genus *Sulfurospirillum*. Focusing on one of the strains, we assessed its chemoautotrophic activities using H_2_ or simple organics like formate as the electron donor, and sulfur or nitrate as the electron acceptor. Through comprehensive physiological, genomic, and comparative genomic analysis, we uncovered unique metabolic characteristics and potential adaptation mechanisms of a novel isolate to SW-HTV environments.

## MATERIALS AND METHODS

### Enrichment and isolation

Sulfur-rich sediments of the Kueishan Island SW-HTV (121.96232°E, 24.83420°N), at depth of 21 m, were used as the inoculum in our study. Detailed sampling information and environmental parameters can be found in our previous studies (41, 42). The surface portion of each sediment core was discarded and the remaining portion was stored in sterile polypropylene bags and kept on ice during transportation to the laboratory.

In the laboratory, 25 g sediment sample was mixed with 20 mL MJ synthetic seawater (44) in a 120 mL serum bottle (Whatman) under a gas phase of pure N_2_ (200 kPa) at 28°C. After 48 h enrichment, 0.5 mL of the sediment slurry was transferred to a new 20 mL MMJHS medium for longer culture under a gas phase of N_2_/H_2_/CO_2_ (50:40:10, 200 kPa). Microbial pure cultures were obtained using a dilution-to-extinction approach. MMJHS medium was prepared by dissolving 1 g each of NaHCO_3_, Na_2_S_2_O_3_∙5H_2_O and NaNO_3_, 3 g S^0^, and 10 mL trace vitamin solution (45) in 1 L of MJ synthetic seawater (44). All the ingredients except sulfur were mixed, and the pH was adjusted to 6.5 with NaOH. Then, the medium was filter-sterilized with 0.2 µm filter (Whatman, United Kingdom). Next, 20 mL medium was distributed into 120 mL serum bottle, which was autoclaved together with sulfur power. Resazurin was added as a redox indicator to monitor bacterial growth. However, we did not add resazurin to the medium during subsequent substrate and growth factor tests to avoid potential disturbances to the optical density (OD) values.

### Substrate test

The spectrum of electron donors and electron acceptors utilized was determined using MJ synthetic seawater containing 0.1% (wt/vol) NaHCO_3_ as the basal medium. Potential electron acceptors, including S^0^ (0.3%, wt/vol), Na_2_S_2_O_3_∙5H_2_O (0.1%, wt/vol), Na_2_SO_3_(0.1%, wt/vol), Na_2_SO_4_ (0.1%, wt/vol), NaNO_3_ (0.1%, wt/vol), and NaNO_2_ (0.1%, wt/vol), were tested individually using hydrogen as the electron donor in a mixed gas phase (N_2_/H_2_/CO_2_ = 50:40:10, 200 kPa). For experiments testing the capability of the isolates to utilize formate (0.1%, wt/vol), fumarate (0.1%, wt/vol), succinate (0.1%, wt/vol), malate (0.1%, wt/vol), pyruvate (0.1%, wt/vol), or lactate (0.1%, wt/vol) as an electron donor, S^0^ (0.3%, wt/vol) was added into the culture medium as electron acceptor, and N_2_ (200 kPa) was provided as the gas phase. Additionally, the ability of the isolates to grow by fermentation on glucose, mannitol, inositol, rhamnose, sucrose, melibiose, amygdalin, and arabinose was tested with API 20E test kits (BiomCrieux, Marcy-I'Etoile, France) under oxygen-free conditions.

To determine nitrogen utilization, 0.10% (wt/vol) NH_4_Cl or NaNO_3_ was added to MMJHS medium lacking of nitrogen sources, under a N_2_/H_2_/CO_2_ (50:40:10, 200 kPa) gas phase. Sulfur utilization was tested by providing S^0^ (0.3%, wt/vol) and/or Na_2_S_2_O_3_∙5H_2_O (0.1%, wt/vol) as the sulfur source to the medium, under a N_2_/H_2_/CO_2_ (50:40:10, 200 kPa) gas phase.

### Morphology and growth factors

The morphology of the cells was examined using fluorescence microscope and transmission electron microscopy. To assess potential growth controlling factors such as pH, temperature, and NaCl concentration, changes in OD at 600 nm were monitored. Sensitivity to oxygen was determined by adding different concentrations of an oxygen scavenger to the medium under the gas phase composed of N_2_/H_2_/CO_2_ = 50:40:10, at 200 kPa. The oxygen scavenger utilized was a mixture of 2.5% Na_2_S·9H_2_O and 2.5% cysteine HCl.

### Analysis of fatty acid, polar lipids, and respiratory lipoquinones

Fatty acid, respiratory quinones, and polar lipids were extracted from 100 mg, 400 mg and 1 g of freeze-dried cells, respectively. Fatty acids were saponified, extracted, and methylated using the standard protocol of MIDI (Sherlock Microbial Identification System, version 6.0) and identified by using the RTSBA6.0 database of the Microbial Identification System (46). Polar lipids of freeze-dried cells were extracted and separated on silica gel 60F254 aluminum-backed thin-layer plates (10 × 10 cm^2^; Merk 5554), which were dried for 30 min at 55°C and further analyzed according to Minnikin et al. (47). The first dimension of the solvent system was chloroform/methanol/water (65:24:4, by volume) and the second dimension was chloroform/glacial acetic acid/methanol/water (80:15:12:4, by volume). Other reagents such as α-naphthol, ninhydrin, and molybdenum blue (Sigma) were used to detect glycolipids, amino lipids, and phospholipids, according to Tindall (48). Phosphomolybdic acid (5%, wt/vol, dissolved in alcohol) was sprayed on the plates, which were then heated at 160°C for 10–15 min, to identify total lipids. Respiratory quinones were extracted using the method described by Minnikin et al. (47) and analyzed by HPLC as described by Tindall (48).

### Molecular analysis

The 16S rRNA gene was amplified via PCR using primers Eubac 27F/1492R (49) and sequenced using Sanger dideoxynucleotide chain-termination method by Sangon Biotech Co. Ltd. (Shanghai, China). Unambiguously nucleotide sequences were used for phylogenetic analysis with MEGA package (50).

Genomic DNA was extracted using MiniBEST bacteria genomic DNA extraction kit Ver.3.0 (Takara BIO INC.) according to the manufacturer’s protocol. The purified genomic DNA was quantified using a TBS-380 fluorometer (Turner BioSystems Inc., Sunnyvale, CA). High-quality DNA (OD260/280 = 1.8–2.0, >20 µg) was sequenced using a combination of PacBio RS II Single MoleculeReal Time (SMRT) and Illumina sequencing platforms by Majorbio Bio-Pharm Technology Co. Ltd. (Shanghai, China). The Illumina data were employed to evaluate the genome complexity. After quality trimming, the reads were assembled into contigs using hierarchical genome assembly process (51). Subsequently, error correction of the PacBio assembly results was performed using the Illumina reads with Pilon. Glimmer (52) was utilized for CDS prediction, tRNA-scan-SE (53) for tRNA prediction, and Barrnap for rRNA prediction. Predicted CDSs were annotated from NR, Swiss-Prot, Pfam, GO (Gene Ontology), COG (Clusters of Orthologous Groups), and KEGG (Kyoto Encyclopedia of Genes and Genomes) databases using sequence alignment tools such as BLAST, Diamond, and HMMER. Each set of query proteins was aligned with the databases, and annotations of best-matched subjects (*e*-value < 10^−5^) were obtained for gene annotation. A phylogenomic tree of Campylobacteria genomes was reconstructed based on 400 universal marker genes using PhyloPhlAn (54).

Genome relatedness was assessed using the ANI (average nucleotide identity) (55), POCP (percentage of conserved proteins), and AAI (average amino acid identity) indexes. ANI value was calculated by pyANI program (56). POCP was calculated as described before (57). AAI was generated using the amino acid identity workflow of CompareM (http://github.com/dparks1134/CompareM).

[FeFe]- and [NiFe]-hydrogenase homologs were discretely distributed at the domain, phylum, and order levels of taxonomic classifications (58). Based on the phylogeny of the large subunits, [NiFe] hydrogenase was further classified into different groups according to a previous study (59). The annotated hydrogenases of our isolates’ genome were aligned with the reference hydrogenase sequences (59) by MUSCLE (60), and a phylogenetic tree was constructed in MEGA (50).

The software was executed with default settings unless other specified.

### Computing the pangenome

The anvi’o pangenome workflow (https://merenlab.org/2016/11/08/pangenomics-v2) was employed to compute the pangenome with default settings (61), involving several steps: (i) generating an anvi’o genome database of *Sulfurospirillum* (anvi-gen-genomes-storage) to store DNA and amino acid sequences (ii), computing the pangenome (anvi-pan-genome) from a genome database by identifying “gene clusters” (iii), displaying the pangenome (anvi-display-pan) to visualize the distribution of gene clusters across genomes and interactively bin gene clusters into logical groups such as “core genes,” “unique genes of 1612,” and “accessory genes absenting in *S. arcachonens* and 1612” bins (iv), reporting summary output as a profile database including the names of gene clusters and their numbers in each bin (anvi-summarize) (v), obtaining the FASTA file with sequences for each bin (anvi-get-sequences-for-gene-clusters), followed by annotation by KEGG or COG. In this context, a “gene cluster” represents sequences of one or more predicted open reading frames grouped together based on their homology at the translated DNA sequence level. Gene clusters with more than one sequence may contain orthologous or paralogous sequences, or both, from one or more genomes analyzed in the pangenome. The figure of pan-genome profile was generated by BPGA with default parameters (62).

### Data description

The assembled circular genomic data of strain 1612 was deposited in NCBI with the accession number CP140614, under an original name “*Sulfurospirillum* sp. 1612.” For genomic comparison, the following reference genomes were utilized: *Sulfurospirillum deleyianum* (DSM 6946^T^, GCA 000024885, 2.3 Mb), *Sulfurospirillum multivorans* (DSM 12446^T^, GCA 000568815, 3.2 Mb), *Sulfurospirillum arcachonense* (DSM 9755^T^, GCA 000597725, 2.7 Mb), *Sulfurospirillum barnesii* (DSM 10660^T^, GCA 000265295, 2.5 Mb), *Sulfurospirillum arsenophilum* (NBRC109478, GCF 000813345, 2.6 Mb), *Sulfurospirillum halorespirans* (DSM 13726^T^, GCA 001723605, 3.0 Mb), *Sulfurospirillum cavolei* (UCH003, GCA 001548055, 2.7 Mb), UCH001 (GCA 001548035, 2.6 Mb), JPD-1 (GCA 002309535, 2.8 Mb), SL2-2 (GCA 002205395, 2.9 Mb), ACSTCE (GCA 011769965, 2.7 Mb), and ACSDCE (GCA 011769985, 2.8 Mb).

## RESULTS

### Enrichment and isolation

A range of temperatures was applied to the cultures, encompassing 28°C, 35°C, 50°C, and 70°C. After several weeks, only the cultures incubated at 28°C showed slight turbidity, indicating probable microbial growth. Consequently, subsequent isolation efforts primarily focused on incubation at 28°C. Eventually, we successfully isolated seven strains, designated as strains 1612, 2216, 34, 4316-2, 3221-6, 1307, and 2301. As depicted in the phylogenetic tree of 16S rRNA gene (Supporting materials, Fig. S1), strains 1612, 34, 2216, 3221-6, and 4326-2 were similar, clustering into one clade, while strains 1307 and 2301 formed another clade. They were genetically related to the genus *Sulfurospirillum*, but the sequence similarity values were not high. For example, strain 1612 exhibited low similarity in its 16S rRNA gene sequence when compared to *Sulfurospirillum carboxydovorans* (91.5%), *S. arcachonense* (93.1%), and the type strain *S. deleyianum* (91.9%) (Supporting materials, Table S1). Strains 1612, 2216, 34, and 1307 were subsequently purified and preserved at the Marine Culture Collection of China (Xiamen, China) with the strain preservation numbers MCCC 1K08293, MCCC 1K08414, MCCC 1K07846, and MCCC 1K08950. Purity confirmation was achieved through microscopic examination and repeated partial sequencing of the 16S rRNA gene using both Sanger sequencing and high-throughput sequencing methods. Strain 1612 was chosen as the representative for subsequent phenomics and genomic analyses due to its faster growth rate.

### Morphology, physiology, and growth characteristics of the isolate

Cells of strain 1612 were observed to be slightly curved rods, with lengths ranging from 1.0 to 2.5 µm, and each cell possessed a polar flagellum (Supporting materials, Fig. S2). The cell was stained Gram-negative. Strain 1612 exhibited growth at 4–50°C, with an optimum growth temperature of 28°C. No growth was observed below 4°C or above 50°C (Supporting materials, Fig. S3A). The growing pH of the bacteria ranged from 5.5 to 8.0, with an optimum growth at pH 6.5 (Supporting materials, Fig. S3B). NaCl requirement for growth was determined by using various concentrations of NaCl (1.0–6.0%, wt/vol) in the medium. The isolate grew in concentration ranging from 1.0% to 5.0% (wt/vol) NaCl, showing optimum growth at 3.0% (wt/vol) NaCl (Supporting materials, Fig. S3C).

The predominant cellular fatty acids identified in strain 1612 were C16:0 (25.19%), C18:1ω (24.67%), and C16:1ω (16.58%) (Supporting materials, Table S2). These profiles were consistent with those observed in most other *Sulfurospirillum* species, except for *S. arcachonense*. The polar lipids detected in strain 1612 included phosphatidylethanolamine (PE) and an unidentified lipids (AL). Two naphthoquinones compounds were identified: menaquinone with six isoprenoid units (MK-6; 4.89%) and monomethylmenaquinone with six isoprenoid units (MMK-6; 95.11%).

### Classification based on phylogenomic analysis

**Fig 1:**
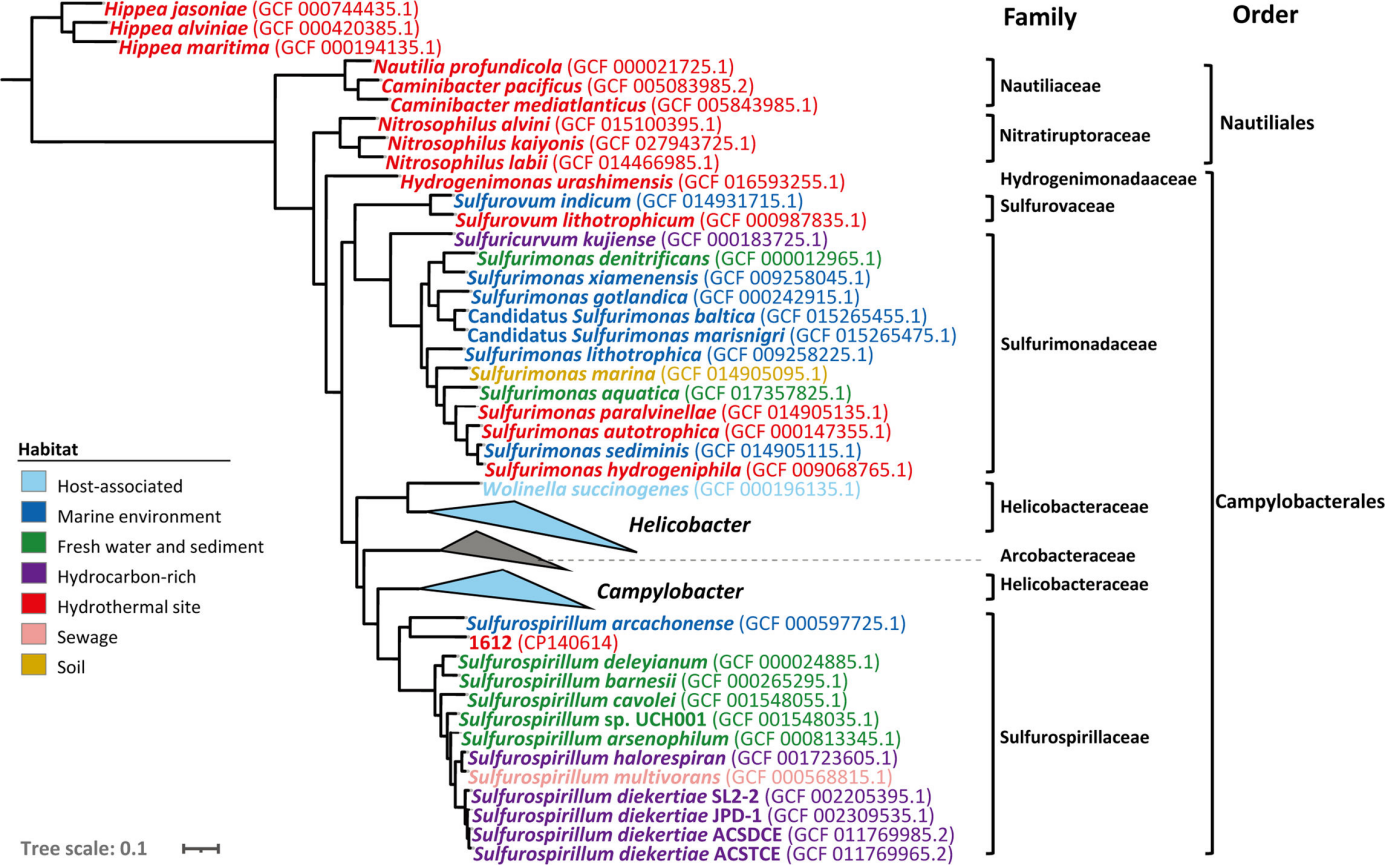
Phylogenomic tree of Campylobacetial isolates. A total of 105 Campylobacetial reference genomes with the complete length were downloaded from NCBI. Combined with some other *Sulfurospirillum* isolates’ genomes, this phylogenetic tree was reconstructed using PhyloPhlAn software. Different colors correspond to the sources of isolates. The root was composed of three genomes of genus *Hippea*. The scale bar indicates 0.1 substitutions per amino acid position.

A phylogenomic tree of existing Campylobacteria genomes was constructed to illustrate the taxonomic relatedness of the isolates (Fig. 1). Notably, *Sulfurospirillum* species are distinct from the other related genera like *Campylobacter*, *Arcobacter*, *Helicobacter*, and *Wolinella*, which are mostly host-associated. In contrast, reported pure cultures of *Sulfurospirillum* have been from diverse environments, including soil, freshwater sediment, sewage, and hydrocarbon-rich sites. In the phylogenetic tree, the newly isolated 1612 formed a clade with *S. arcachonense*, which was isolated from marine sediment.

We compared the ANI, AAI, and POCP values among the genomes of genus *Sulfurospirillum*. The ANI values between 1612 and other *Sulfurospirillum* genomes were around 71% (Supporting materials, Fig. S4; Table S3), far below the hypothesized species demarcation threshold of 95% (63), indicating that 1612 represents a distinct species. The ANI value (Fig. S4; Table S3) was also lower than the threshold for genus definition (73.98%) (64), but AAI and POCP values were around the threshold to assign 1612 into a novel genus (Supporting materials, Fig. S4; Table S4). The 63–65% of AAI value between 1612 and other strains (Supporting materials, Fig. S4; Table S4) was below the suggested 65% threshold to set a novel genus (65), but a little higher than the suggested 60% boundary mentioned in another study (66). The results of POCP values (55–63%, in Supporting materials, Table S5) were higher than 50%, a genus boundary for prokaryotic lineages (57). Considering the uncertainty of POCP value in delineating a new genus, we proposed strain 1612 to be a novel species within the genus *Sulfurospirillum*, named as *Sulfurospirillum kueishanense* sp. nov. However, the potential to establish a new genus out of genus *Sulfurospirillum* needs to be considered in the future.

### Genomic features of the novel Campylobacteria isolate from SW-HTVs

The genome of strain 1612 (accession no. CP140614) comprised one circular chromosome with 2,377,931 bp containing 2365 open reading frames (Supporting materials, Table S6; Fig. S5). The total length of all the identified genes was 2,228,148, accounting for 93.7% of the total genomic length. The total GC-content was 37.84%. A total of 6 rRNAs (5S + 16S + 23S) and 39 tRNAs were detected. Approximately 90.19% of all identified genes, about 2,133 genes, could be annotated by the COG database. Excluding “function unknown,” the categories “energy production and conversion” and “amino acid transport and metabolism” were the most abundant, represented by about 227 and 200 genes, respectively (Supporting materials, Fig. S6). Using the KEGG database, approximately 58.01% of all identified genes, about 1372 gene, could be annotated. Most of the annotated genes were related to “metabolism” pathway (green histogram in Supporting materials, Fig. S7).

The genome of 1612 contained three genomic islands (Supporting materials, Table S6), termed GI01 (coordinates 1,478,760–1,492,033, total 13,273 bp), GI02 (coordinates 2,140,982–2,183,349, total 42,367 bp), and GI03 (coordinates 97,646–133,215, total 35,569 bp), possibly acquired via horizontal gene transfer. Most of the genes in the genomic islands encoded hypothetical proteins, but some appeared to encode the functional proteins related to the adaption mechanisms to the HTVs’ environment. For example, the GI02 included the formate transporter (gene2204, *fdhC*) and formate hydrogenlyase (FHL) complex (gene2205 to gene2214) (Supporting materials, Table S7), one of the hydrogenases identified in 1612.

### Metabolisms and potential functions of the Campylobacteria isolate from SW-HTVs

**Fig 2:**
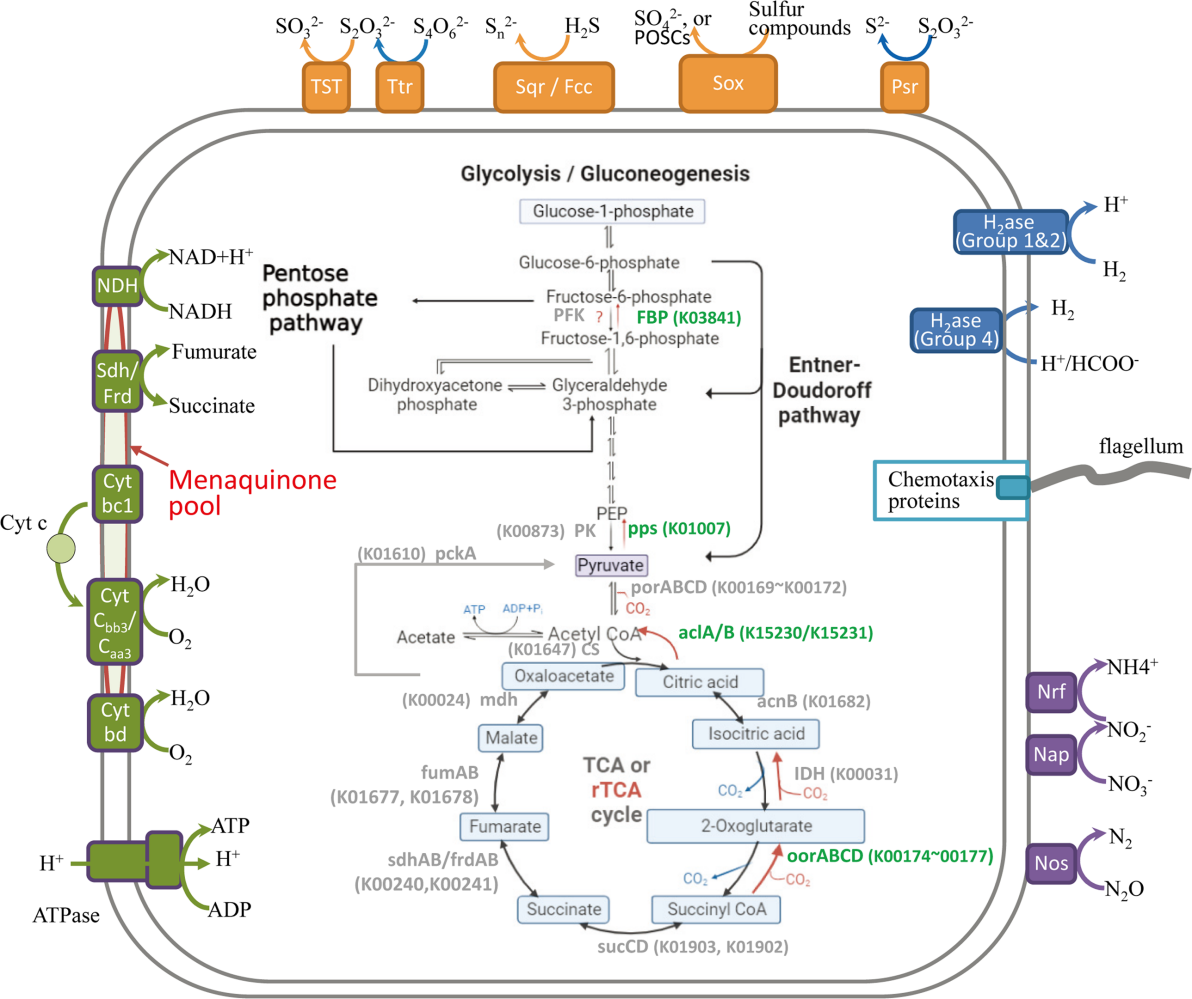
Overview of the central metabolism pathways in strain 1612. The presence and absence (marked with question marks) of genes were predicted based on the annotations from KEGG. Created via BioRender.com. H_2_ase, hydrogenase; Nap, periplasmic nitrate reductase; Nrf, nitrite reductase; Nos, nitrous oxide reductase; TST, thiosulfate sulfurtransferase; Ttr, tetrathionate reductase; Sqr, sulfide-quinone oxidoreductase; Sox, sulfur-oxidation multienzyme; Psr, thiosulfate reductase; PFK, phosphofructokinase; FBP, Fructose 1,6-bisphosphate; pps, phosphoenolpyruvate synthase; PK, Pyruvate kinase.

By combining genome-scale analyses with known physiological traits, the metabolic network of strain 1612 was reconstructed (Fig. 2). The result showed that the bacteria may live a chemolithoautotrophic lifestyle with high plasticity on metabolisms, potentially contributing to several essential biogeochemical processes such as carbon fixation, hydrogen oxidation, sulfur reduction/oxidation and nitrate reduction.

Carbon fixation in strain 1612 mainly occurs via the rTCA cycle (reductive citric acid cycle, or reductive tricarboxylic acid cycle), which reverses the reactions of the oxidative citric acid cycle (TCA cycle) and forms acetyl-CoA from two CO_2_ molecules (Fig. 2). Characteristic enzyme genes of the rTCA cycle, including fumarate reductase (*frdAB*), 2-oxoglutarate synthase (*oorABCD*), ATP-citrate lyase (*aclAB*), and pyruvate:ferredoxin oxidoreductase (*porABCD*), were all identified in the genome of strain 1612 (Fig. 2). The initial steps of gluconeogenesis pathway, forming fructose-6-phospahte from oxaloacetate or pyruvate (KEGG module M00003), were complete in 1612.

In addition to carbon fixation, strain 1612 also showed potential for using organic carbon. While glycolysis was incomplete due to the lack of some irreversible enzymes (such as phosphofructokinase in catalyzing phosphorylate fructose-6-phosphate to fructose-1,6-bisphosphate), the potential to degrade glucose was supplemented by the Entner-Doudoroff pathway (KEGG module M00008) in 1612.

Adenosine triphosphate (ATP) production in strain 1612 occurs through substrate-level phosphorylation and oxidative phosphorylation. Substrate level phosphorylation (KEGG module M00579) involves enzymes like phosphate acetyltransferase (Pta) producing acetyl-phosphate from acetyl-coenzyme A, and enzyme acetate kinase (AckA) producing acetate from acetyl-phosphate. Additionally, strain 1612 possesses an F-type ATPase to synthesize ATP through oxidative phosphorylation, utilizing a series of enzyme complexes in the respiratory chain and menaquinone to shuttle electrons along the electron transfer chain. The detected genes of respiratory complexes included the Complex I (NADH:quinone oxidoreductases I, *nuoA ~N*), Complex II (Succinate dehydrogenase/Fumarate reductase, *sdhBC* and *frdABC*), Complex III (Cytochrome bc1 reductase), Complex IV (Cytochrome *caa_3_*-type oxidase, *coxABCD*; and the *cbb3*-type, *ccoNOPQ*), and the Cyt bd oxidoreductase (*cydAB*) (Fig. 2). Both *cbb_3_*-type cytochrome c oxidase (67) and Cyt bd oxidoreductase (68) have a high affinity for oxygen and typically function under low-oxygen conditions. In contrast, cytochrome *caa_3_*-type oxidase has a low affinity for oxygen and is usually expressed and functional under high oxygen conditions in many bacterial species, such as *Paracoccus denitrificans*, *Bradyrhizobium japonicum*, and *Rhodobacter sphaeroides* (69–71).

**Fig 3:**
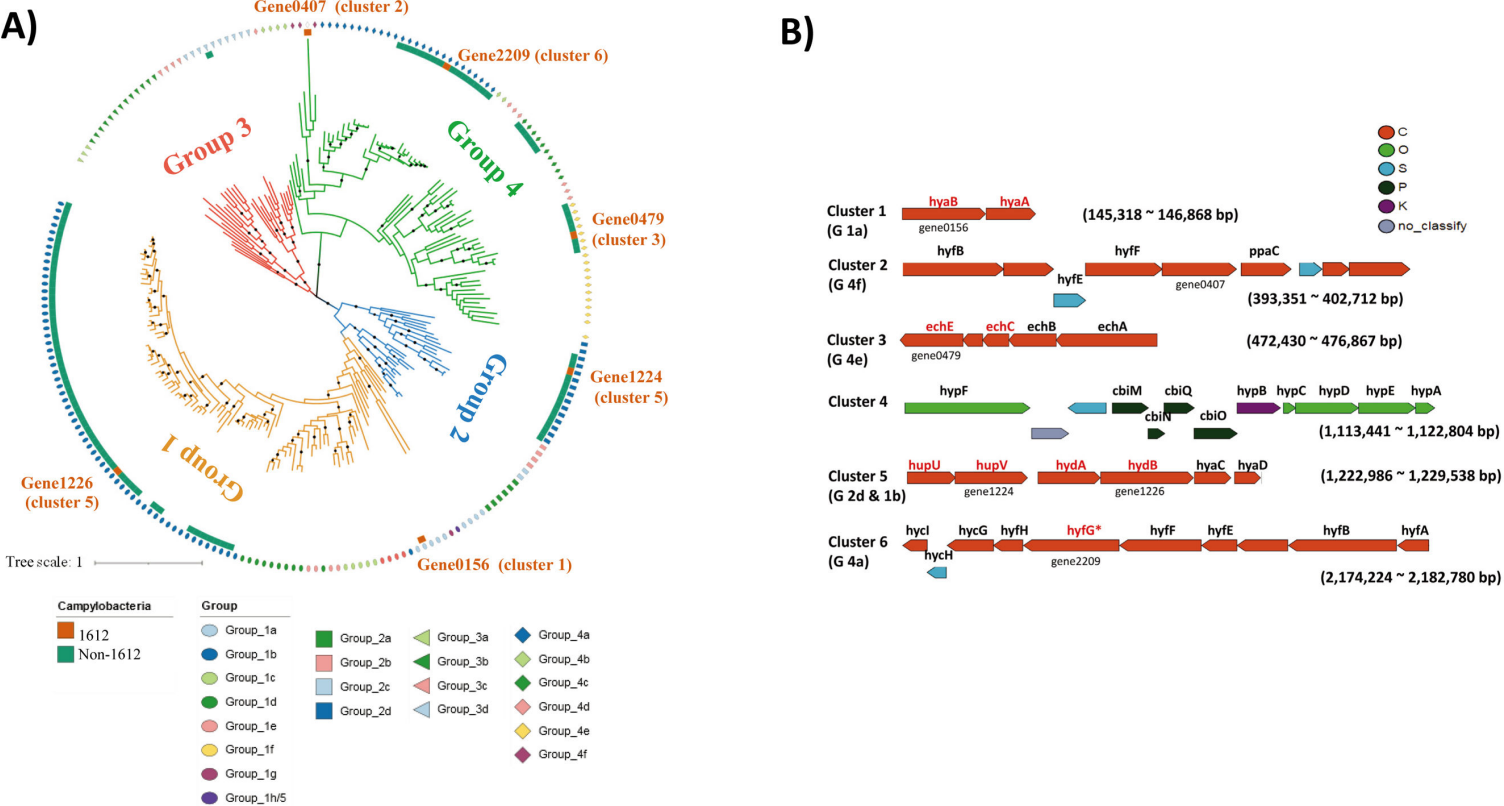
Classification and phylogeny of hydrogenases identified from 1612 genome. (**A**) The neighbor-joining skeleton trees showed the phylogenetic relationships of hydrogenases structural gene large subunit identified from strain 1612 and previously reported bacteria (59). The central circle trees were colored by [NiFe]-hydrogenase subgroups. The black nodes indicated the well-supported nodes (bootstrap values >80). Detailed information was shown in Fig. S9; (**B**) The six hydrogenase clusters identified from the genome of 1612. The color of genes corresponded to the COG categories. The gene names of structural genes of hydrogenase were colored in red. The large subunit used to construct the phylogenetic tree was marked with a gene number. Each cluster included one or two groups of hydrogenases. The hydrogenase subgroups of each cluster were shown in the brackets below the cluster name. The gene *hyfG* of cluster 6 was annotated as *hycE* in KEGG, but we renamed it to “*hyfG*” as it belonged to group 4a hydrogenase in the phylogenetic tree.

Hydrogenase catalyzes reversible H_2_ oxidation, H2↔2H^+^+2e^−^ (72). In strain 1612, five [NiFe]-hydrogenase gene clusters and one hydrogenase accessory *hyp* gene cluster were detected (Fig. 3B). Based on the constructed phylogenetic tree, [NiFe]-hydrogenase detected in strain 1612 were identified as subgroup 1a, 1b, 2d, 4a, 4e, and 4f (Fig. 3A). The hydrogenase structural gene large subunit of subgroup 1a and 4f, corresponding to gene0156 and gene0407 in 1612, was not found in other Campylobacteria (Fig. 3A), indicating the presence of diverse hydrogenase in strain 1612.

Strain 1612 also possesses genes encoding enzymes involved in oxidizing (Sqr, Sox, Ttr, and TST) or reducing (Psr) sulfur compound (Fig. 2). It also included genes for dissimilatory nitrate reduction to ammonium (DNRA) (such as *nap* and *nrf* genes involving in reducing nitrate and nitrite reduction to ammonium) and part of denitrification pathway (*nos* gene involving in N_2_O to nitrite and dinitrogen), suggesting a role in nitrogen cycling.

### Physiological experiments validating the metabolic capabilities of strain 1612

**Fig 4:**
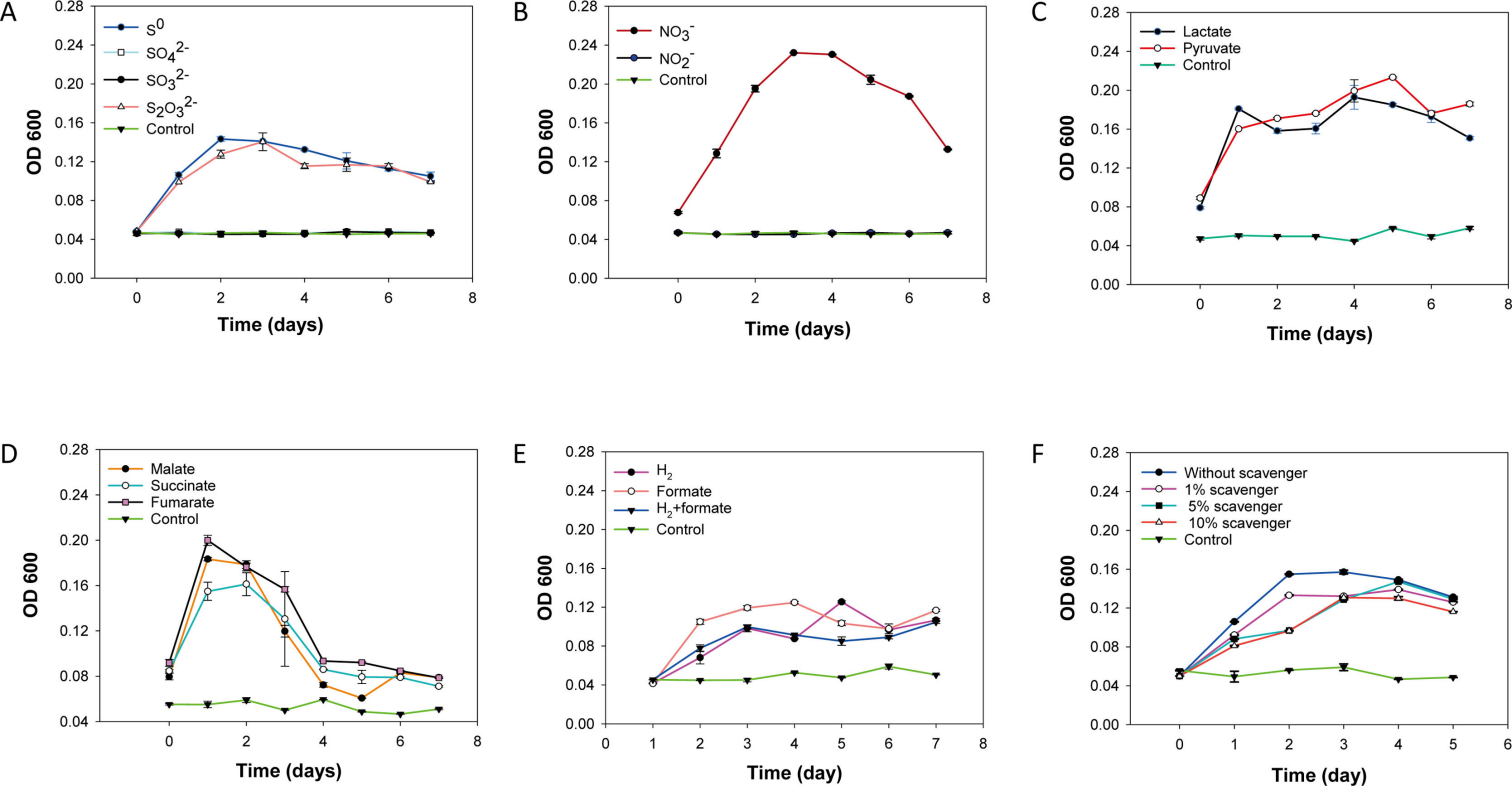
The growth curves for the substrate testing. The spectrum of electron acceptor testing when using H_2_ as the sole electron donor (A&B). The electron donor testing was proceeded by adding lactate, pyruvate, fumarate, succinate, malate, or formate when S^0^ was the sole electron acceptor (C–E). The growth profile of 1612 under different concentrations of oxygen scavenger (**F**). All the “control” in the figure were referred to the negative control, meaning without bacterial inoculum. The electron donor and electron acceptor of the negative control for each testing were listed in pairs: H_2_/S^0^ (**A**), H_2_/NO_3_^−^ (**B**), lactate/S^0^ (**C**), malate/S^0^ (**D**), H_2_/S^0^ (**E**), and H_2_/(S^0^, S_2_O_3_^2^−, NO_3_−) (**F**).

The physiological experiments performed aimed to validate the annotated chemotrophic lifestyle of the isolated Campylobacteria species. The results demonstrated that strain 1612 exhibited robust growth when utilizing CO_2_ as the sole carbon source in conjunction with H_2_ as the electron donor, and either nitrate, thiosulfate or S^0^ as electron acceptors (Fig. 4A and B). However, no growth was observed when sulfate, sulfite or nitrite was employed as electron acceptors in conjunction with hydrogen as the sole electron donor and CO_2_ as the only carbon source (Fig. 4A and B). Strain 1612 also displayed the ability to utilize simple organic carbon sources, including formate, lactate, pyruvate, fumarate, succinate, and malate, as the electron donors (Fig. 4C, D and E). Each test was conducted with a single type of organic carbon added, while S^0^ served as the sole electron acceptor and CO_2_ as the only carbon source. Interestingly, when acetate was added, strain 1612 exhibited growth on formate as the sole electron donor in conjunction with nitrate, thiosulfate, or S^0^ as the sole electron acceptors (Supporting materials, Fig. S8A and B). Moreover, strain 1612 appeared growing better in the MMJHS medium containing both S^0^ and S_2_O_3_^2−^ (Supporting materials, Fig. S8D). However, no growth was observed when sucrose was used as electron donor and S^0^ as the electron acceptor (data not shown). The sensitivity to oxygen in strain 1612 was also tested. As shown in Fig. 4F, strain 1612 was not a strict anaerobe and can tolerate a certain level of oxygen, as evidenced by better growth in the absence of an oxygen scavenger.

### Pan-genome analysis and functional traits for bacterial adaptation in SW-HTVs

**Fig 5:**
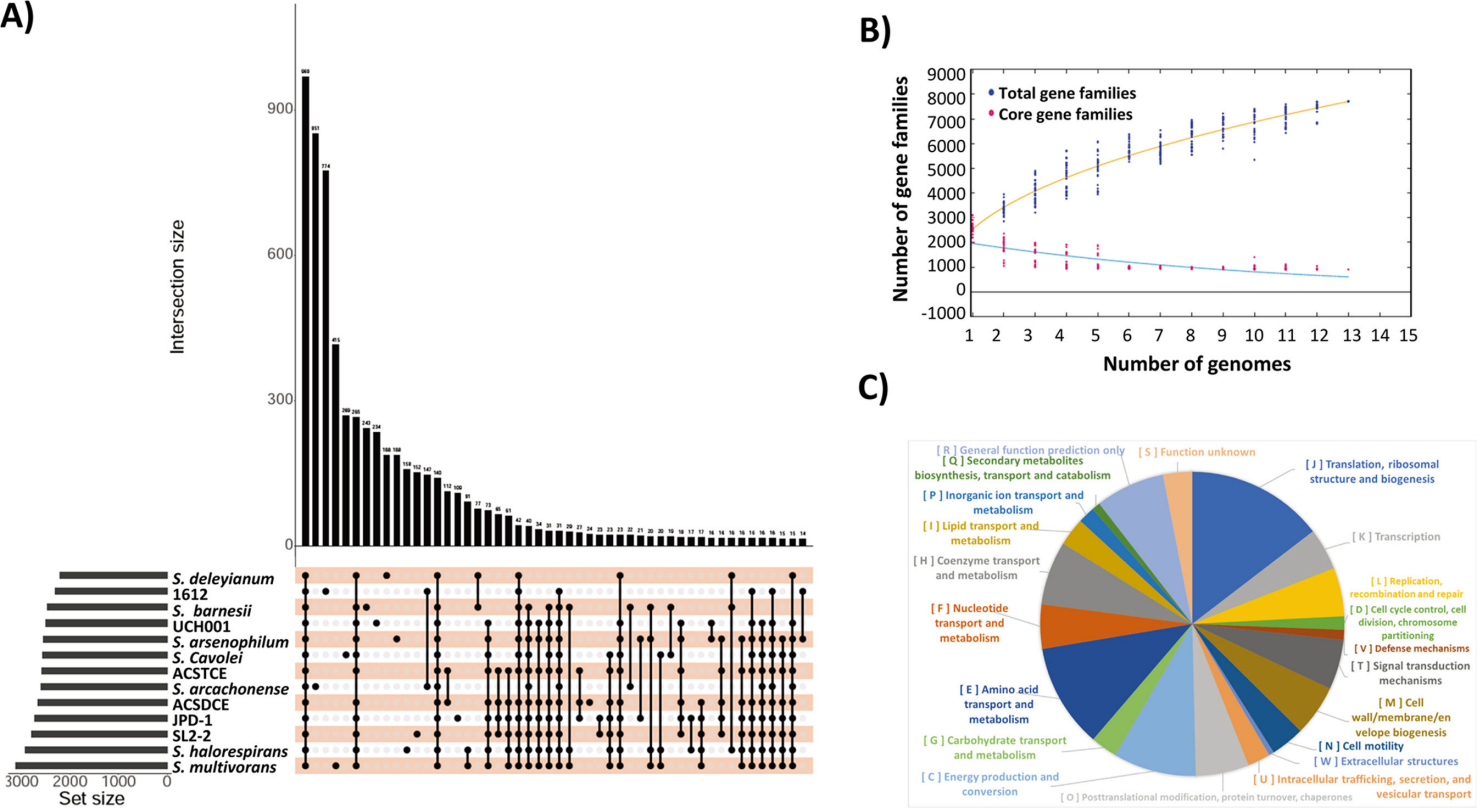
Pan-genome analysis results. (**A**) Upset plot to highlight the number of genes sharing among genomes. The variable component of genomes contributing to a collection of gene clusters was marked by black dots. (**B**) The Pan-genome profile trends of the genus *Sulfurospirillum*. (**C**) The COG annotation results for the shared core genes.

We analyzed 13 complete genomes of *Sulfurospirillum* strains, including the newly isolated 1612 in this study. These strains were sourced from diverse origins. All the genomes were utilized to construct the genomic phylogenetic tree (Fig. 1). The anvi’o pan-genomic pipeline distinguished 7,553 gene clusters (GCs) out of a total of 35,485 genes across the 13 genomes. The highest numbers of GCs were observed in the core genes shared among all genomes, followed by the unique genes of *S. arcachonense*, 1612, *S. multivorans*, and *S. cavolei* (Fig. 5A). Most of the core genes were associated with essential functions, such as energy production/conversion, translation, and amino acid transport/metabolism (Fig. 5C). The pan-genome profile illustrated that as the number of sequenced genomes increased, the pan-genome size expanded rather than reaching a plateau (Fig. 5B). Consequently, it can be inferred that the pan-genome of the genus *Sulfurospirillum* followed an open type, indicating a strong potential for horizontal gene transfer events. Considering the potential of 1612 to be a novel genus, we recalculated the pan-genome profile of genus *Sulfurospirillum* without including strain 1612. The result still indicated an open-type pan-genome (Supporting materials, Fig. S10).

**Fig 6:**
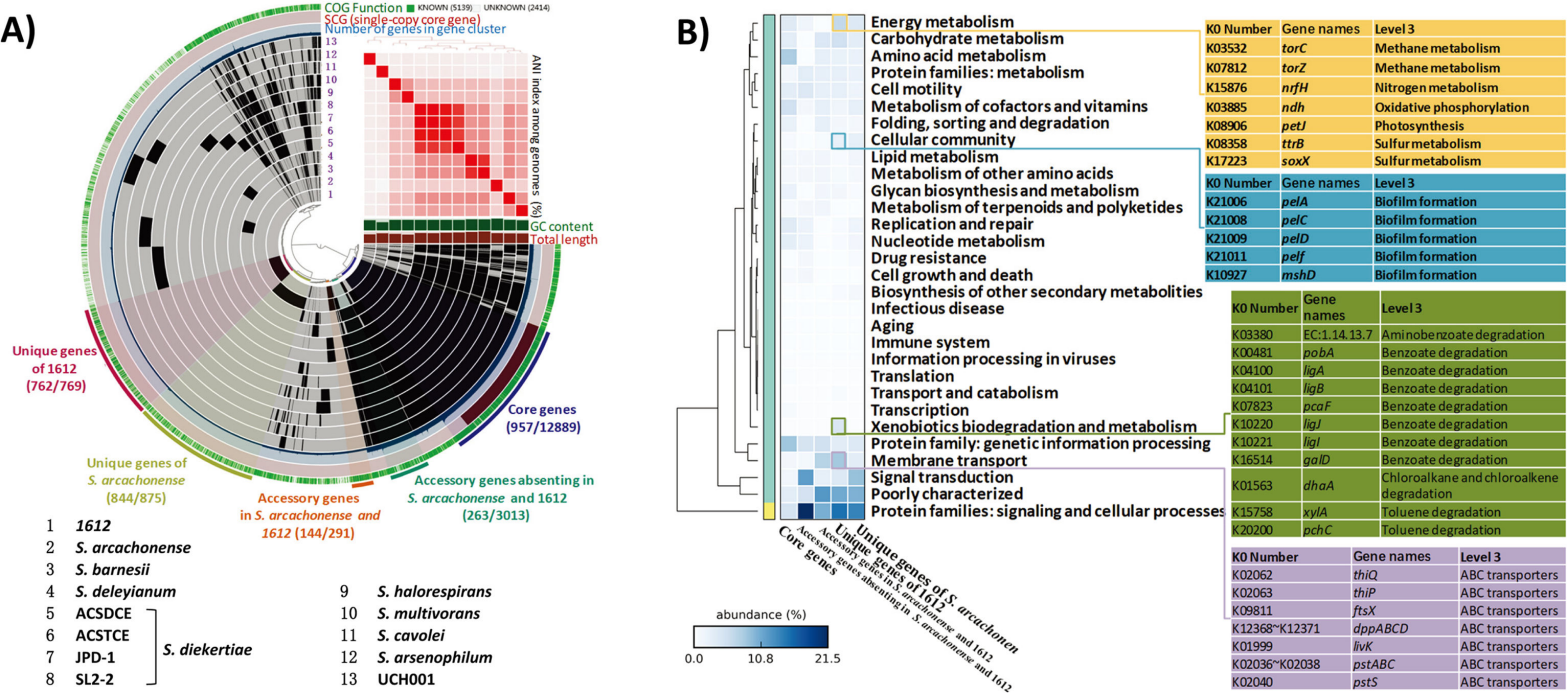
Pan-genome analysis for 13 *Sulfurospirillum* genomes using the visualization platform of Anvi’o. (**A**) The first 13 layers, in which dark color indicated the presence of a gene cluster and light color indicated its absence, represented individual genomes. ANI values among different genomes were represented on heatmap determined from the high similarity (red) and low similarity (white). Five specific groups of gene clusters were selected into bins such as the “core genes,” which were indicated in different colors in the outmost layer. The numbers of gene clusters and responding genes included in each bin were listed under the bin names, and separated with “/.” The dendrograms on the top represented the hierarchical clustering of genomes based on the frequencies of gene clusters. (**B**) The breakdown of gene annotation results shows the component of different KEGG level 1 categories in total gene numbers for each selected bin. Genes without annotated information were not shown in the figure but used in the calculation of relative abundance. Some annotation categories of “unique genes of 1612” bin were extended to show the information of its unique KOs.

To discern the functional distinction between 1612 and other strains, we identified five bins including the core gene bin, two accessory gene bins, and two unique genes bins using the visualization platform of the Anvi’o pan-genomic workflow (Fig. 6A). The “core genes” comprised 12,889 genes belonging to 957 GCs (Fig. 6A). Most of its genes could be annotated by KEGG (79%) or COG (96%), while the ratio of annotated genes was much lower for the other bins (Supporting materials, Table S8). The GCs number of the “unique genes of 1612” bin and “unique genes of *S. arcachonense*” bin was comparable to the “core genes” (Fig. 6A). Protein families involved in “protein families: signaling and cellular processes,” “signal transduction,” “membrane transport,” and some “poorly characterized” genes had higher ratios in the unique genes of 1612 and *S. arcachonense* compared to the core genes (Fig. 6B). At a deeper functional annotation level, signal-related processes including “transporters,” “two-component system,” and “ABC transporters” were much more prevalent in the unique and accessory genes than the core genes (Supporting materials, Fig. S11). At the gene level, we also analyzed the KEGG orthology (KO) detected only in 1612. It was found that 1612 had specific ABC transporters of dipeptide, phosphate, and thiamine (Fig. 6B, purple block). Furthermore, several genes related to biofilm formation and benzoate/toluene degradation were exclusively detected in the unique genes of 1612 (Fig. 6B, blue and green blocks). Regarding energy metabolism, some genes associated with sulfur, nitrogen, methane, oxygen, and hydrogen were only detected in the “unique genes of 1612” (Fig. 6B, orange block, supporting materials, Table S9).

The annotation results of COG were also carefully analyzed between the “unique genes of 1612” and “core gene” bins. The category “P” related to inorganic ion transport and metabolism was significantly higher in “unique genes of 1612” than in “core gene” bins (Supporting materials, Fig. S12B). A total of 72 COGs showed significant differences between these two bins (Supporting materials, Table S10). For “unique gene of 1612,” a significantly higher abundance of COGs was found in metabolism-related categories (such as P, C, and G). Specifically, rhodanese-related sulfurtransferase (COG0607 and COG2897), cytochrome c nitrite reductase (COG3303), Fe (COG4773 and COG1918), and phosphate (COG0226, COG0581, and COG0573) were abundant in the category “P.” In the category “C” related to energy production and conversion, hydrogenlyase (COG0650, COG0651, and COG5557), cytochrome c_553/551/552_ (COG2863 and COG4654), and *napC* (COG3005) were notably abundant. Additionally, the category “G” associated with carbohydrate transport and metabolism exhibited higher abundance in TRAP-type C4-dicarboxylate (e.g., succinate, fumarate, l-malate, and d-malate) transport system (COG1593 and COG3090), glycosyltransferase (COG1216), glycogen synthesis (COG0297 and COG0296), and alpha-amylase/alpha-mannosidase, GH57 family (COG 1449) (Supporting materials, Table S10; Fig. S13).

### Comparison of key energy metabolic genes

**Fig 7:**
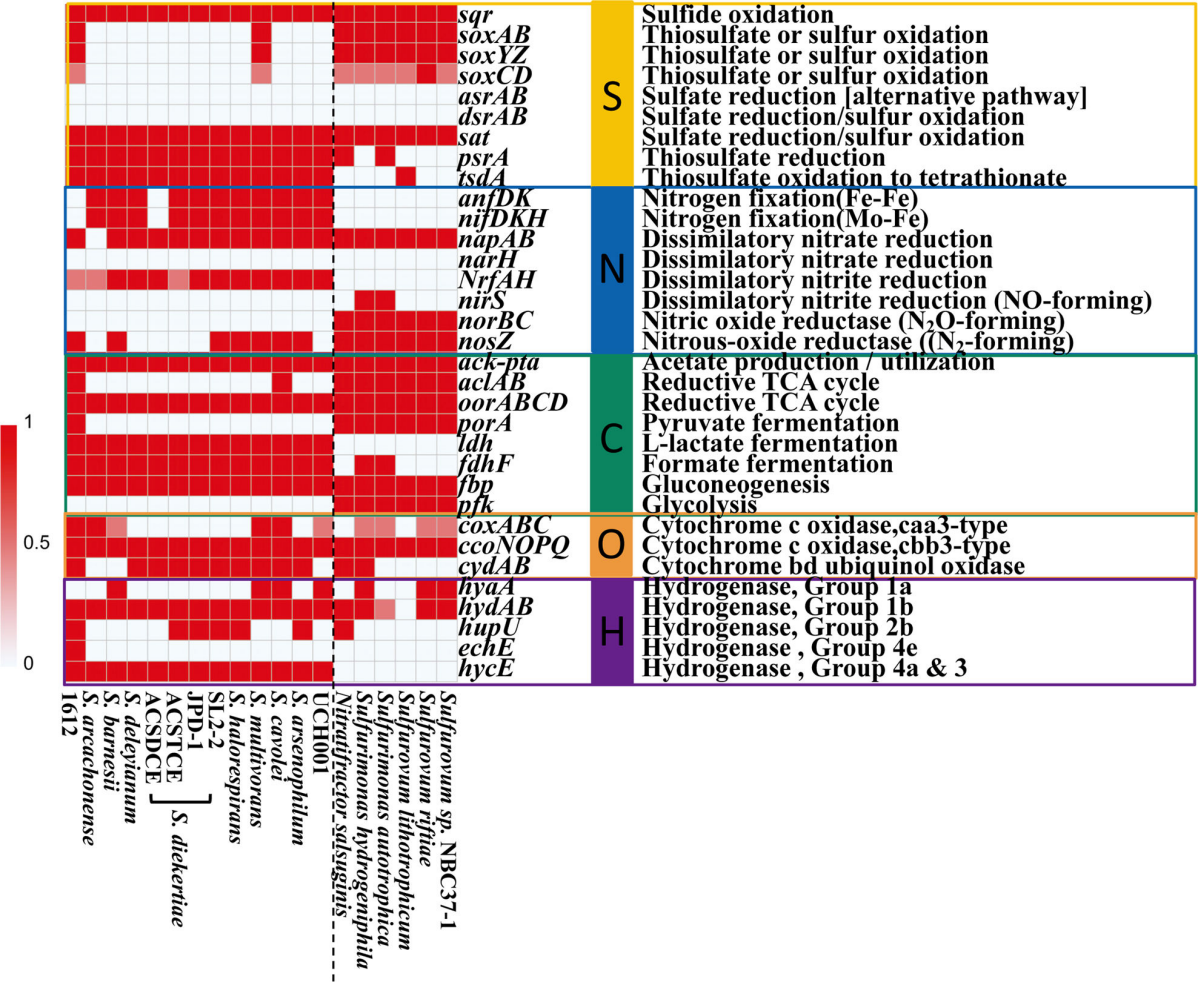
Color-coded table indicating major functional genes and their abundance in 13 genomes of *Sulfurospirillum* and 6 genomes of mesophilic chemolithotrophic Campylobacteria. The *y*-axis indicated the genes likely involved in energy metabolism, and the *x*-axis indicated the strain designations. Red color corresponded to the complete genes identified in carbon, sulfur, and nitrogen nutrient cycles; pink indicated the partial identified in the gene cluster; white indicated an absence of relevant genes.

Genes involved in energy metabolisms were compared among genomes, including 13 genomes of *Sulfurospirillum* and 6 genomes of mesophilic Campylobacterial isolates from deep-sea HTVs (Fig. 7). The genome of strain 1612 harbored characteristic enzyme genes of rTCA cycle for CO_2_ fixation (encoded by genes *aclAB* and *oorABCD*), sulfur oxidation system SOX, hydrogenase, and nitrate reductase, supporting the potential of chemoautotrophy. The genes of CO_2_ fixation (*aclAB*) and sulfur oxidation system (*soxABCDYZ*) were presented in the genomes of all collected isolates from deep-sea HTVs and 1612. Except for 1612, the potential of rTCA cycle for CO_2_ fixation (*aclAB*) was also detected in *S. cavolei*, but was absent in other *Sulfurospirillum* species.

## DISCUSSION

Our novel isolate 1612 from the Kueishan Island SW-HTVs enriched the species diversity of Campylobacteria. The two major orders, Nautiliales and Campylobacterales, within Campylobacteria, exhibited clear differences in their metabolic potential and core bioenergetics (6). Genera belonging to the order Nautiliales, such as *Nautilia*, *Caminibacter*, *Lebetimonas*, *Cetia*, *Nitrosophilus*, and *Nitratiruptor*, are commonly found in deep-sea HTVs, according to data from LPSN (List of Prokaryotic names with Standing in Nomenclature). In contrast, most cultured representatives of the order Campylobacterales thrive at lower temperatures and can typically tolerate a much greater range of oxygen concentrations (15). Genera *Sulfurovum* and *Sulfuromonas* are the frequently studied Campylobacterales in vent area, with few reports of genera *Nitratifractor* and *Hydrogenimonas*, based on data from LPSN. Although the initial enrichments of Campylobacteria isolated from deep-sea HTVs (including Am-H and Ex-18.2) (73) are recognized as *Sulfurospirillum* and have been tested to be moderately thermophilic sulfur-reducing heterotrophs or sulfur lithoautrotrophs using hydrogen as the electron donor, their draft genomes do not provide a basis for deeper comparison with other typical strains of *Sulfurospirillum*. In this study, our novel isolate (strain 1612) of genus *Sulfurospirillum* expands the strain diversity of Campylobacterales in hydrothermal vent areas. Through systematic genomic and phenotypic study, these results may contribute to a better understanding of mesophilic Campylobacteria in hydrothermal vent systems.

The ability to fix CO_2_ was only detected in strain 1612, but not in other *Sulfurospirillum* species. Genus *Sulfurospirillum* consists of versatile, often microaerophilic bacteria, capable of growing with various growth substrates (74), including hydrogen, formate, nitrate, sulfur compounds, and many toxic compounds (e.g., PCE, toxic arsenate, and selenate). Upon thorough comparison of phenotypic and genomic features within genus *Sulfurospirillum*, we found that the capacity for chemoautotrophy appears to be a unique metabolism for the novel *Sulfurospirillum* strain from the Kueishan Island SW-HTVs (strain 1612) (Table 1; Fig. 7). Although chemoautotrophic potential has been partially shown by detection of the *aclAB* gene in the genome of *S. cavolei* (75), our study is the first to clearly demonstrate the chemolithoautrophic capacity of the genus *Sulfurospirillum* via combined results of genomic features and experimental validations. Similar to the prevalent Campylobacterial isolated from deep-sea HTVs (76), such as the mesophilic *Sulfurovum* and *Sulfurimonas*, chemolithoautotrophy also appears to be a common feature of Campylobacteria in SW-HTVs. On the other side, considering the unique ability to fix CO_2_ in genus *Sulfurospirillum* and the monophyly formed by strain 1612 and *S. arcachonense* in the phylogenomic tree (Fig. 1), the potential to establish strain 1612 and our other isolates into a new genus out of genus *Sulfurospirillum* needs to be considered in future studies.

**TABLE 1:** Differential phenotypic characteristics of 1612 and other *Sulfurospirillum* species^*b*^^,^^*c*^

Characteristics	1	2	3	4	5	6	7	8	9	10
Morphology	Slightly curved rods	Curved spiral rods	Curved/ helical rods	Vibrioid to spirillum	Vibrioid to spirillum	Vibrioid to spirillum	Slightly curved rods	Vibrioid to spirillum	Slightly curved rods	Vibrioid to spirillum
Width (µm)	0.3–0.6	0.3–0.5	0.6	0.3	0.3	0.3	0.6	0.5–0.7	0.4–0.5	0.3
Length (µm)	1.0–2.5	1.0–3.0	2.5–4.0	1.0–2.5	1–2	1–2	2.5–4.0	1.2–2.5	1.5–5.0	2.0–3.0
Presence of flagella	+	+	+	+	+	+	+	+	+	+
Colony color	Whitish	Yellow	ND	Whitish	ND	ND	ND	ND	ND	Greyish
Chemoautotrophy	+	−	−	−	−	−	−	−	−	−
Growth rage (optimum)										
Temperature (°C)	20–37 (28)	20–36 (ND)	ND (30)	8–30 (26)	ND (33)	ND (20)	ND (25–30)	ND	20–40 (30)	9.9–31.9 (30.2)
NaCl (%)	2.0–4.0 (3.0)	<0.2 (ND)	ND (ND)	0.6–4.0 (1.2–2.0)	ND (0.05)	ND (0.1)	ND (ND)	1.3–3.5 (1.3)	<1.0 (0)	1.0–5.5 (2.0)
pH	5.5–8.0 (6.5)	ND (7.0–7.1)	ND (7.0–7.5)	6.1–8.2 (7.0–7.4)	ND (7.5)	ND (7.5)	ND (ND)	7.1–9.7 (8.5)	6.0–8.0 (7.0)	5.9–8.5 (7.3)
Electron acceptors										
Nitrate	+	+	+	−	+	+	+	+	+	+
Nitrite	−	+	−	−	+	+	+	+	−	+
Oxygen (microaerobic)	+^*a*^	+	ND	+	+	+	+	−	+	+
S^0^	+	+	ND	+	+	+	+	+	+	+
Sulfite	−	+	−	−	−	ND	−	ND	+	+
Thiosulfate	+	+	−	−	+	+	−	+	+	+
Sulfate	−	−	−	−	ND	ND	−	ND	−	ND
Arsenate	ND	−	+	−	+	+	+	+	+	+
PCE	−	ND	+	ND	−	ND	+	ND	−	+
Selenate	ND	−	+	ND	+	−	+	−	−	+
DMSO	ND	+	ND	−	−	ND	ND	−	+	+
Electron donors
Hydrogen	+	+	+	+	+	+	+	+	+	+
Formate	+	+	+	ND	+	+	+	+	+	+
Fumarate	+	+	+	+	+	+	ND	+	+	+
Succinate, malate,	+	+	ND	+	ND	ND	ND	ND	+	+
Pyruvate	+	+	+	+	+	+	+	+	+	+
CO	ND	ND	ND	−	ND	ND	ND	ND	ND	+
Lactate	+	−	+	+	+	+	+	+	+	+
Major quinone	MK-6 and MMK-6	MK-6 and MMK-6	MK	MK-6 and MMK-6	ND	ND	ND	ND	ND	ND
Isolated from	Sulfur-rich sediment of shallow-water HTVs	Mud of forest pond	Activated sludge	Surface sediment of an intertidal mud flat	Freshwater marsh	Freshwater marsh	Soil	Bioreactor	Groundwater storaging crude oil	Marine sulphide-rich seep
DNA G + C content (mol%)	37.8	40.6	41.5	32	40.8	40.9	41.8	47.6	42.7	30.5
Gene bank accession no.	CP140614	GCA_000024885	GCA_000568815	GCA_000597725 (Draft)	GCA_000265295	GCF_000813345 (Draft)	GCA_001723605	ND	GCA_0015 48055	ND

^*a*^ Strain 1612 is a facultative aerobe. Its sensitivity to oxygen is evaluated by a simple test involving the addition of oxygen scavenger to the medium (results see Fig. 4F).

^*b*^ Strains: 1, 1612 (this study); 2, *S. deleyianum* DSM 6946^T^ (77, 78); 3, *S. multivorans* DSM 12446^T^ (79, 80); 4, *S. arcachonense* DSM 9755^T^ (81); 5 and 6, *S. barnesii* DSM 10660^T^ and *S. arsenophilum* DSM 10659^T^, respectively (82); 7, *S. halorespirans* DSM 13726^T^ (80); 8, *S. alkalitolerans* DSM 24537^T^ (83); 9, *S. cavolei* DSM 18149 (75); and 10, ‘*S. carboxydovorans’* DSM 16295 (84). +, positive; −, negative; ND, not data. All strains are able to utilize the following as electron donors: formate (+ acetate), hydrogen (+ acetate) and pyruvate. All strains are positive for the fermentation of fumarate.

^*c*^ Abbreviations: MK, Menaquinon; MMK, monomethylmenaquinone.

Another important functional feature of strain 1612 is the presence of diverse subgroups of [NiFe] hydrogenases in its genome. Three major classes of hydrogenase, featured with a [NiFe], a [FeFe], or a [Fe] core, have been identified. Based on the phylogeny of the large subunits, [NiFe] hydrogenases are further classified into four groups: the membrane-bound and cytosolic H_2_-uptake [NiFe] hydrogenases (Group 1 and Group 2), bidirectional heteromultimeric cytosolic [NiFe]-hydrogenases (Group 3), and membrane-bound H_2_-evolving [NiFe] hydrogenases (Group4) (85). In strain 1612, the detected phylotypes of [NiFe]-hydrogenase belong to subgroups 1b, 2d, 4a, 4c, 4e, 1a, and 4f (Fig. 3A), suggesting that both H_2_-uptaking and evolving potentials present in the bacterium.

Hydrogen oxidation is widely distributed in Campylobacteria (58). As demonstrated in *Wolinella succinogenes* (86), most Campylobacteria only contain Group 1b hydrogenase genes (58). The newly isolated strain 1612 harbors both subgroup 1a, 1b and cytosolic H_2_-uptake subgroup 2d hydrogenase (Fig. 3). Subgroup 2d hydrogenase inferred to generate reductant for carbon fixation via rTCA (87). The observed diverse phylotypes of H_2_ utilization hydrogenase genes might be essential for strain 1612’s survival in hydrothermal vent ecosystem, as hydrogen oxidation is among the chemosynthetic reactions providing the greatest energy yields (88). Moreover, hydrogen oxidation seems to be a favorable energy source for autotrophic carbon fixation compared to the oxidation of sulfide/thiosulfate, despite considerably more energy being yielded through the oxidation of sulfide/thiosulfate than through hydrogen oxidation (free standard enthalpies are −797 kJ/mol H_2_S vs −237 kJ/mol H_2_ with O_2_ as electron acceptor) (89). This is because the redox potential of hydrogen is more negative than that of the reducing equivalent NAD(P)/H; in contrast to sulfide, a reverse electron transport is not required in conjunction with hydrogen oxidation. Thus, only a third of the energy is required for fixing 1 mol of carbon when oxidizing hydrogen compared to sulfide (1,060 kJ for hydrogen vs 3,500 kJ for sulfide) (90).

The Group 4a (*Hyf*) and its homologous Group 3 (*Hyc*) hydrogenase both function in the FHL complex during fermentation, which produces H_2_ in *Escherichia coli* (85). Similar with Group 4e (*Ech*) [NiFe]-hydrogenase, they are absent in genomes of typical Campylobacteria strains isolated from deep-sea HTVs, such as those belonging to genera *Sulfurovum*, *Sulfuromonas*, and *Nitratifractor* (Fig. 7). In contrast, the existence of *hyf* genes (Group 4a) have been reported in the most genomes of *Sulfurospirillum*, such as *S. multivorans* (91) and *Sulfurospirillum diekertiae* (92). The H_2_ production activities have also been demonstrated in *Sulfurospirillum* species, that is, *S. multivorans*, *S. cavolei*, *S. deleyianum*, and *S. arsenophilum*, which can fermentative grow on pyruvate and produce H_2_ after about 20 generations of transfer from the initial cultures (93). The similar structures of *hyf* gene clusters between strain 1612 and *Sulfurospirillum* species (93) support the possibilities of H_2_ production in strain 1612, yet the detailed mechanisms and *in-situ* activities of H_2_ production need to be further studied in the future.

Diverse respiratory oxygen reductases (terminal oxidases) are found in Campylobacteria isolated from SW-HTVs. In vent areas, Campylobacteria occupy high-sulfide, low-oxygen niche compared to chemoautotrophic Gammaproteobacteria (94, 95). Considering the sharp chemical and physical gradients formed during the mixing of reduced hydrothermal fluids with oxic seawater, the importance of oxygen for Campylobacteria is underscored. Recent short-term incubations have indicated the importance of oxygen in structuring natural communities and affecting carbon fixation efficiency for Campylobacteria (96). There are three enzyme superfamilies capable of acting as terminal respiratory oxygen reductases—heme-copper oxygen reductases, alternative oxidases, and Cyt bd oxidoreductases (97). Most terminal oxidases belong to the heme-copper oxygen reductases superfamily, such as cytochrome c oxidase (Cox), which uses a c-type cytochrome as its electron donor. Cox emzyme are universal in aerobic and facultative aerobic organisms, represented by *caa_3_*-type and *cbb_3_*-type oxidases (98). For our strain 1612 isolated from SW-HTVs, genes for respiratory oxygen reductases with both high (*cbb_3_*-type Cox and Cyt bd oxidoreductase) and low (*caa_3_*-type Cox) affinity for oxygen were identified in its genome (Fig. 2). This indicates that strain 1612 is a facultative aerobe, a conclusion supported by the physiological test with oxygen scavenger addition (Fig. 4F). In comparison, most isolates of genera *Sulfurovum* and *Sulfurimonas* from deep-sea HTVs only have the genes *ccoNOPQ* of *cbb_3_*-type Cox (Fig. 7). Higher concentrations of oxygen are reported in SW-HTVs than in their deep-sea counterparts (4, 99). This may be due to more effective mixing with oxygen-rich surface waters and the influence of tidal and wave actions. In Kueishan Island, high temporal variations in water temperature are attributable to diurnal tides (100). It is, therefore, reasonable to expect a similar variation in other parameters within SW-HTVs (99). The presence of more types of oxygen reductases in strain 1612 confirms the higher variable in oxygen concentration in SW-HTVs compared to the deep-sea environment.

Through our study, we identified unique genes in strain 1612 by comparing its genome with the ones of non-vent strains of *Sulfurospirillum* (Fig. 6B). These unique functional traits of strain 1612, possibly gained via genome extension events (e.g., gene horizontal transfer). Indeed, at least three genomic islands in genomes of 1612 (Supporting materials, Table S6) were found to correspond to the presence of horizontal gene transfer events. Such genomic plasticity increases micro-diversity and confers a competitive advantage enabling this lineage to thrive in changing physical–chemical gradients of hydrothermal systems (101). Reflecting the complex environmental conditions within SW-HTVs, the diverse signal genes identified in unique genes of strain 1612 may facilitate its adapting and dominance in vent area. This finding prompts us to focus on specific sources (e.g., dipeptide, phosphate, and thiamine) within the vent area to gain a deeper understanding of Campylobacteria. However, due to limitations in genome numbers and functional annotation, the results still lack a refined selection of target genes. Further physiological experiments involving transcriptome analysis and gene knockout techniques are necessary to elucidate the details of the adaptation mechanisms.

### Description of *Sulfurospirillum kueishanense* sp. nov

*Sulfurospirillum kueishanense* (kuei.shan.en’ se. N.L. neut. adj. *kueishanense*, pertaining to the Kueishan Island, the western Pacific Ocean, the locality from which the strain was isolated). Slightly curved cells are 0.3–0.6 µm wide and 1.0–2.5 µm long. Gram-negative. Motile by polar flagellum. Colonies are whitish and round. The pH range is 5.5–8.0; optimum, 6.5. The temperature range is 20–37°C; growth optimum, 28°C. The salinity range is 2.0–4.0% NaC1; optimum growth occurs in the presence of 3.0% NaCl. Chemolithoautotrophy. Uses thiosulfate (incomplete reduction), sulfur, and nitrate as electron acceptors. Utilizes formate and H_2_, pyruvate, and fumarate as electron donors. MK-6 and MMK-6 are the sole quinones, with MMK-6 predominating. The predominant fatty acids in the membrane lipids include C_18:1_ ω*7c*, C_16:0_, and C_18:0_, with seldom C_13:0_ and C_15:1_. The GC content of the genomic DNA is 37.8 mol%. The type strain is 1612 (MCCC 1K08293). Isolated from the sulfur-rich sediment of SW-HTVs (Kueishan Island, western Pacific).

## SUPPLEMENTAL MATERIAL

Supplemental figures msystems.00148-24-s0001.docx Fig. S1 to S13.

Supplemental tables msystems.00148-24-s0002.xlsx Tables S1 to S10.

ASM does not own the copyrights to Supplemental Material that may be linked to, or accessed through, an article. The authors have granted ASM a non-exclusive, world-wide license to publish the Supplemental Material files. Please contact the corresponding author directly for reuse.
