# Supplementary material for: Novel isolates of hydrogen-oxidizing chemolithoautotrophic Sulfurospirillum provide insight to the functions and adaptation mechanisms of Campylobacteria in shallow-water hydrothermal vents
Source: mSystems. 2024 Aug 21;9(9):e00148-24. doi: 10.1128/msystems.00148-24 (PMC11406935; doi:10.1128/msystems.00148-24)
Supplement: Supplemental figures — Fig. S1 to S13. [file msystems.00148-24-s0001.docx]

**Figure S1** Phylogenetic tree of 16S rRNA gene sequences showing the position of our isolated strains. Numbers of nodes represent levels of bootstraps support (%) based on a Neighbor-joining analysis of 500 resampled datasets. GeneBank accession numbers were given in parentheses. Bar, 0.02 changes per nucleotide position. One cluster included 1612, 34, 2216, 3221-6 and 4326-2 (the last three strains owing100% similarity with each other but 99.9% similarity with strain 34 or 1612). Another cluster was formed by strains 1307 and 2301, owing a similarity of 99.8% with each other. The similarity between strain 2301 and its closest strains was less than 95%, such as *S. carboxydovorans* (94.0%), *S. arcachonense* (94.4%) and type strain *S. deleyianum* (90.4%) (Supporting materials, Table S1).


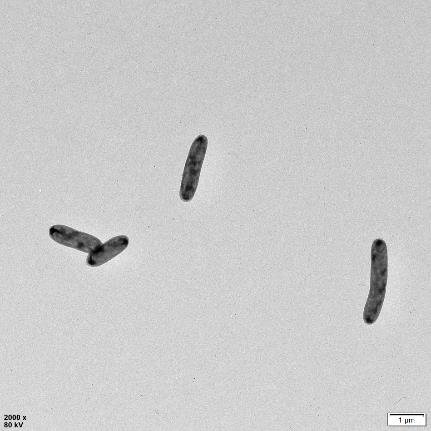

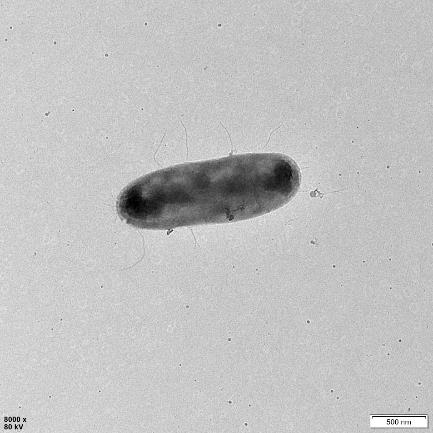




**Figure S2** Transmission electron micrograph of cells of 1612. Bar, 1um (left) or 500 nm (middle, right).

**Figure S3** The growth curves for the tests of temperature (A), pH (B), salinity (C), electron acceptors when using formate (D&E, also added with acetate) as the sole electron donor, and the kinds of N source (F).

**
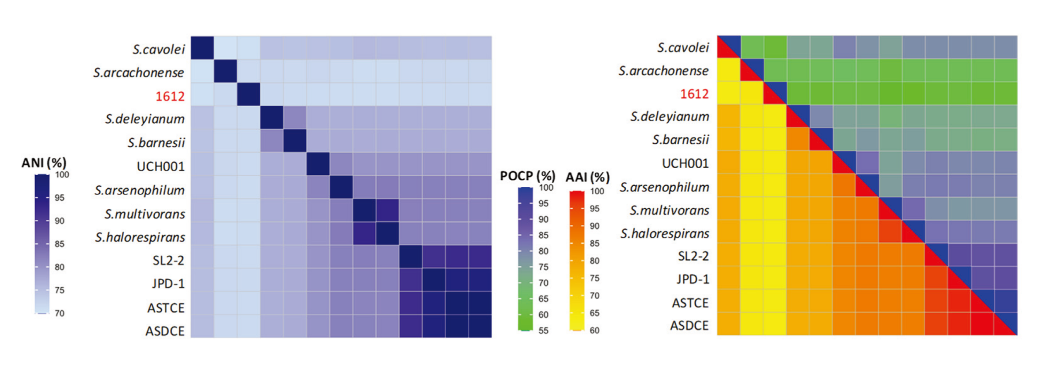
Figure S4** The heatmaps of ANI, AAI and POCP values among the typical genomes of *Sulfurospillum*.


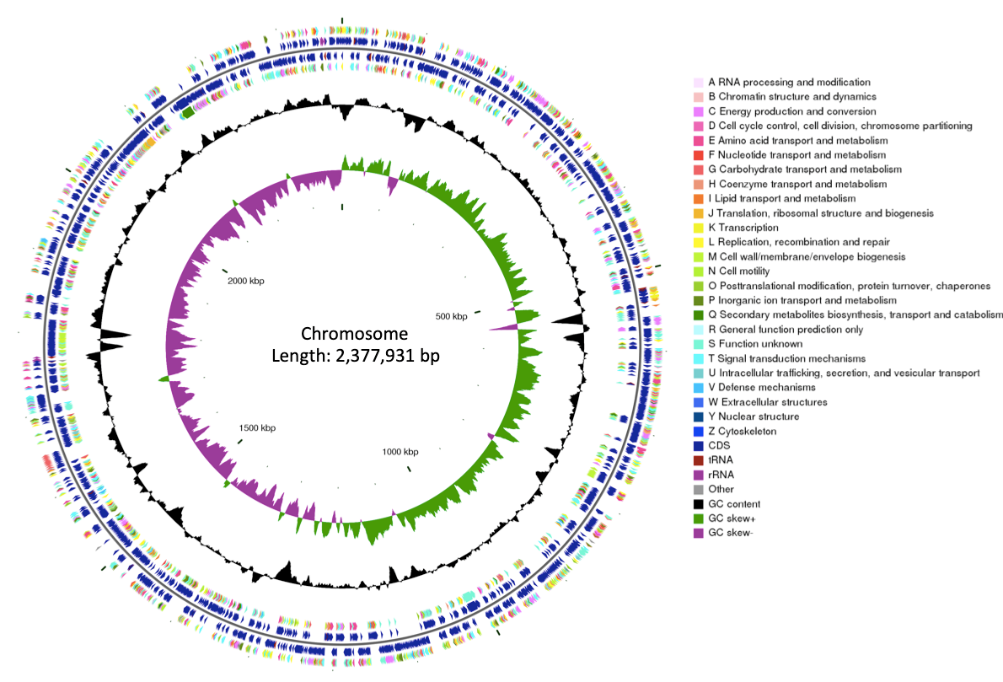


**Figure S5** Graphical circle map of the 1612 chromosome. From the inside to the outside, the first circle represents the scale; the second circle represents the GC skew; the third circle represents the GC content; the fourth and seventh circles represent the COG annotation categories with different colours; the fifth and sixth circles represent the position of CDs, tRNA, and rRNA in the genome.


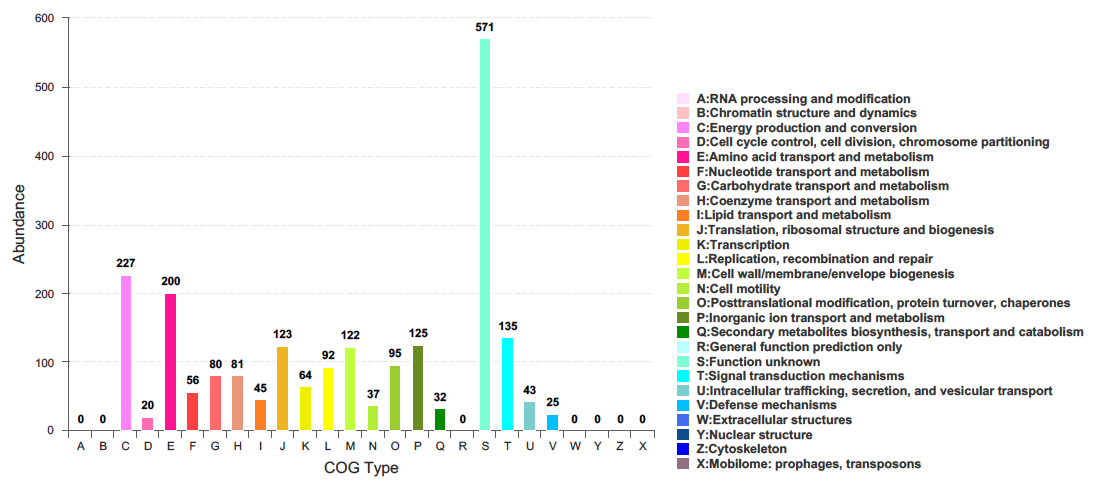


**Figure S6** COG function classification of 1612. The X-axis corresponded to COG categories, while the Y-axis was the number of genes foreach category. The description of every COG category was in the right legend.


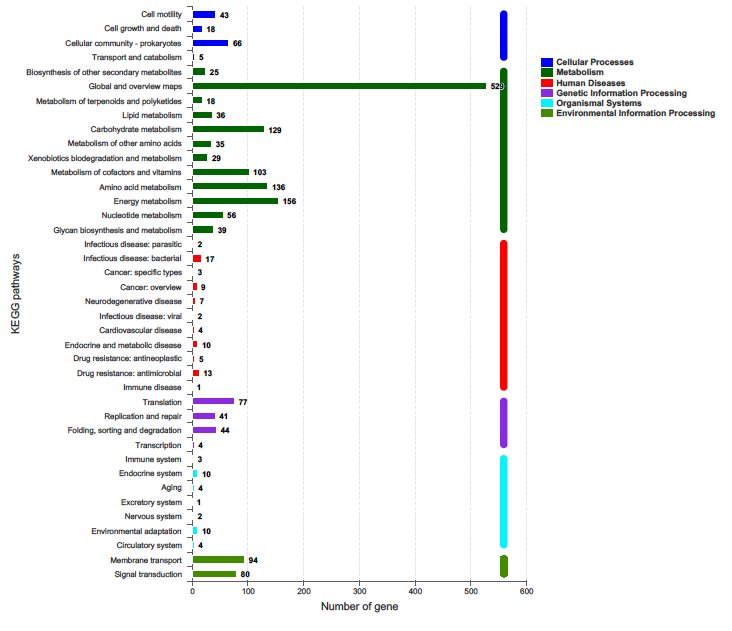


**Figure S7** KEGG annotation results. The level 2 categories of KEGG pathway were shown in the Y-axis. Different color of the histogram corresponded to the level 1 categories.

**Figure S8** The growth curves for the tests of electron acceptors when using formate (A&B, also added with acetate) as the sole electron donor, and the kinds of N source (C) and S source(D).


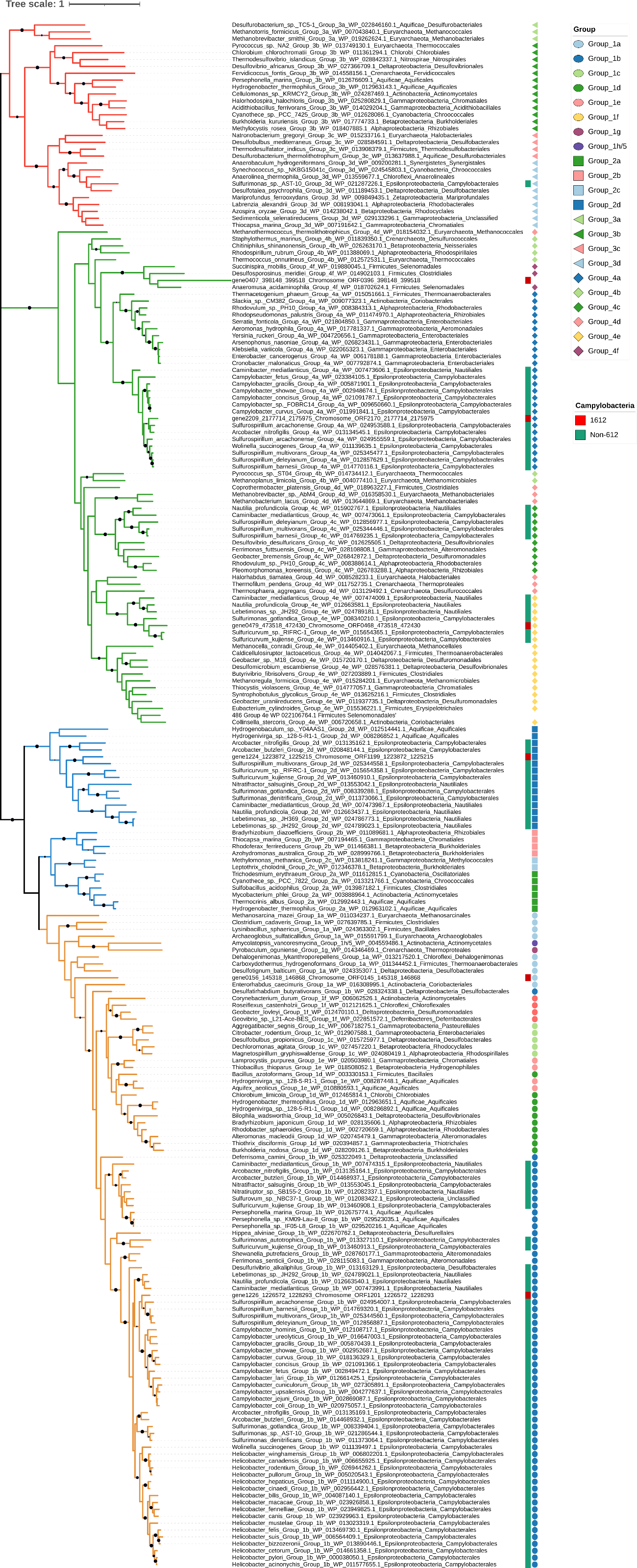
**Figure S9** Phylogenetic tree showing the evolutionary relationships between all [NiFe]-hydrogenases. Branches and taxa are colour-coded based on their subgroup classification. This tree shown the detail information of each sequence in Fig. 3.

 **Figure S10** The Pan-genome profile trends of the genus *Sulfurospirillum* without the genome of strain 1612. Similar with Fig. 5B, the pan-genome size became larger and larger with the number of sequenced genomes increased, instead of tending to a plateau.


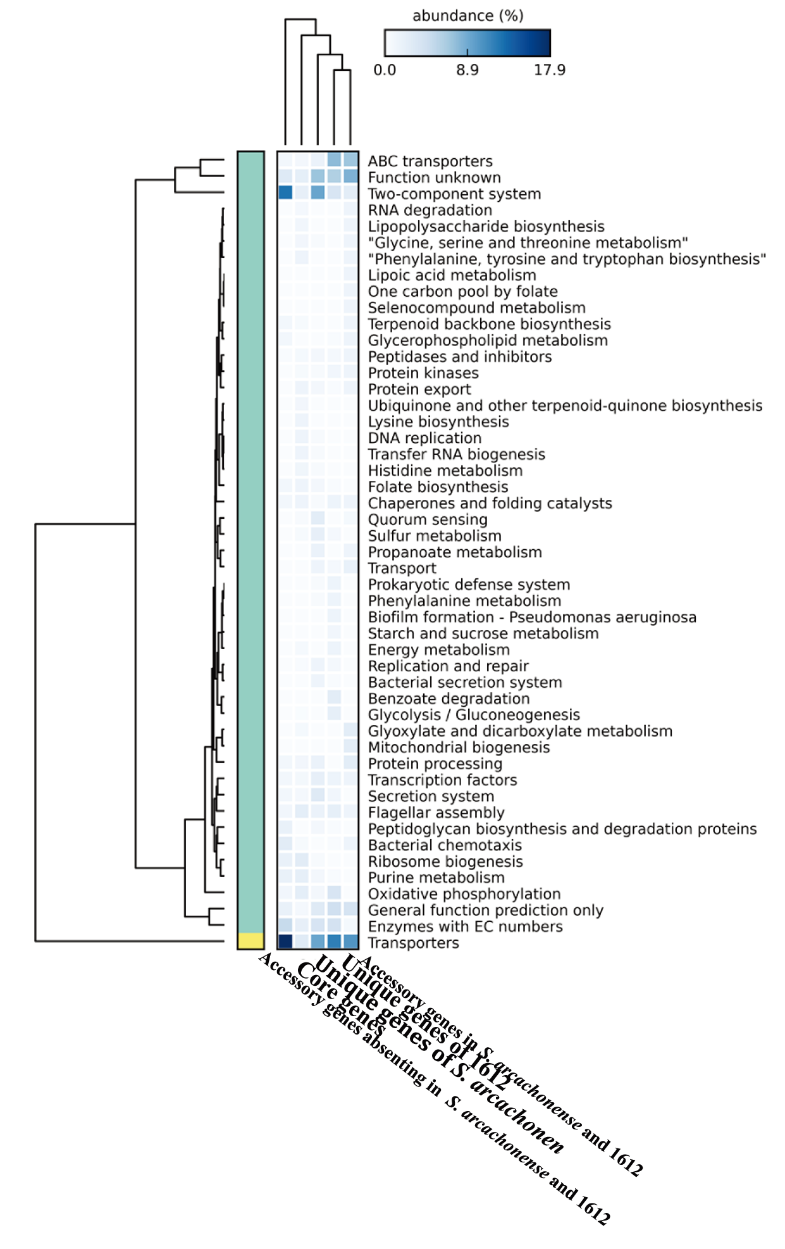
**Figure S11** The breakdown of gene annotation results to show the component of different KEGG level 3 categories for each selected bin. Categories with >1% relative abundance of total genes in each bins were shown. Genes without annotated information was not shown in the figure but used in the calculation of relative abundance.

**Figure S12** The breakdown of gene annotation results by COG database (A) and extended error bar plot for the categories that have a significant difference between any two pangenome bins (B,C,D). Genes without annotated information was not shown in the figure as “others”.

**Figure S13** The extended error bar plot for the COGs that have a significant difference between any two pangenome bins and own more than 3 genes.
